# Large herbivores in novel ecosystems - Habitat selection by red deer (*Cervus elaphus*) in a former brown-coal mining area

**DOI:** 10.1371/journal.pone.0177431

**Published:** 2017-05-15

**Authors:** Anke Müller, Maria Dahm, Peder Klith Bøcher, Meredith Root-Bernstein, Jens-Christian Svenning

**Affiliations:** 1 Department of Bioscience, Section for Ecoinformatics & Biodiversity, Aarhus University, Aarhus, Mid-Jutland, Denmark; 2 Study Faculty Landscape Architecture and Landscape Planning, Technical University Munich, Freising-Weihenstephan, Bavaria, Germany; University of Alberta, CANADA

## Abstract

After centuries of range contraction, many megafauna species are recolonizing parts of Europe. One example is the red deer (*Cervus elaphus*), which was able to expand its range and is now found in half the areas it inhabited in the beginning of the 19^th^ century. Herbivores are important ecosystem engineers, influencing e.g. vegetation. Knowledge on their habitat selection and their influence on ecosystems might be crucial for future landscape management, especially for hybrid and novel ecosystems emerging in post-industrial landscapes. In this study, red deer habitat selection was studied in a former brown-coal mining area in Denmark. Here, natural settings were severely changed during the mining activity and its current landscape is in large parts managed by hunters as suitable deer habitat. We assessed red deer habitat preferences through feces presence and camera traps combined with land cover data from vegetation sampling, remote sensing and official geographic data. Red deer occurrence was negatively associated with human disturbance and positively associated with forage availability, tree cover and mean terrain height. Apparently, red deer are capable of recolonizing former industrial landscapes quite well if key conditions such as forage abundance and cover are appropriate. In the absence of carnivores, human disturbance, such as a hunting regime is a main reason why deer avoid certain areas. The resulting spatial heterogeneity red deer showed in their habitat use of the study area might be a tool to preserve mosaic landscapes of forest and open habitats and thus promote biodiversity in abandoned post-industrial landscapes.

## Introduction

Abandoned industrial areas such as former coal mining sites, sand and gravel pits, stone quarries, suburban landfills and extracted peatlands are becoming ever more common in today’s anthropogenic landscapes [1]. In fact, they are just one example of a current land abandonment phenomenon throughout all of Europe [2], where no-longer profitable land uses are given up, raising the question of how these leftover landscapes should be managed. There are currently two ways of managing the wastelands that are left behind after industrial activity ceases: the sites may either receive some technical reclamation e.g. by adding a layer of fertile topsoil and re-cultivation into agricultural fields, meadows or forests, or they are left to be recovered by spontaneous succession [3]. Especially when allowing for spontaneous succession, these sites may develop so-called novel ecosystems. Ecosystems are called novel if “[…] systems have been potentially irreversibly changed by large modifications to abiotic conditions or biotic composition” [4]. Especially in mining sites, abiotic conditions are often severely changed [5] with e.g. a completely altered topography after resource excavations, soils that are contaminated with heavy metals or a changed hydrologic balance as often ground water tables were lowered during mining activities but rise again after the pumping down stops, forming new lake landscapes. Notably, the highly modified environmental conditions at abandoned industrial sites often result in species compositions that diverge from those in natural ecosystems. Nevertheless, these ecosystems may still serve as valuable habitats for many species [6–9]. As they become increasingly common in our Anthropocene age, their value for nature conservation and the need for protection has been advocated [10, 11]. The various successional stages that often develop on former industrial sites if spontaneous succession is allowed have been shown to enhance plant [12], invertebrate [13] and bird [1] diversity because of the heterogeneity of the resulting landscapes. Especially early successional stages favour habitat specialists that are often rare in our anthropogenic landscapes [1].

In large parts of Europe, forest cover has varied between closed and relatively more open over time [14–16]. During the historical period, humans cleared more and more areas using them for agriculture, livestock farming and building of settlements, shaping the open landscapes one nowadays experiences nearly all over Europe. Increasingly, traditional land use forms like grazing livestock are no longer economical for farmers [2, 17]. Where traditional land uses decline, red deer and other wild herbivores might be keystone species for shaping contemporary ecosystems. Red deer has indeed been shown to hinder forest regeneration [18] and it is also capable of keeping landscapes in open states by slowing down or even prohibiting the succession of shrubs and trees [19]. Understanding and taking advantage of the behaviour of large wild herbivore populations in post-industrial and post-agricultural areas might actually be one solution to maintain heterogeneous landscapes where light, open nature areas occur intermixed with forests without relying on human interventions and at relatively low costs [20].

The red deer *(Cervus elaphus)* is one of the most widely distributed free-roaming large herbivore species in Europe. It is an ecosystem engineer that affects living conditions of other species through its effects on vegetation, mainly by grazing and trampling [20–22], epizoochory [23] and it is capable of keeping landscapes in fairly open states [24]. In Europe, red deer populations declined during the 16^th^ -19^th^ centuries due to overhunting, forest loss and competition with domestic livestock. Through changes in legislation, hunting practice and habitat availability red deer has nowadays recovered in many European countries [25]. Studies have shown several factors determining habitat selection by red deer. First of all, habitat selection is determined by fodder quantity and quality with vegetation composition being the most important factor for the selection of a certain feeding habitat [18]. Red deer prefer species-rich grasslands [26] and forest gaps with a high abundance of herbaceous plants or forbs [27, 28]. Other high quality forage often preferred by deer includes young deciduous trees [18] and deciduous shrubs [28], both also often found in forest clearings and early successional states. Red deer will adjust to lesser energy intake by having greater home range sizes [28]. Other environmental factors influencing habitat choice can cause them to use habitat patches with less desirable fodder quality. For example, deer have been found to have a preference for young dense coniferous stands, especially in winter. This vegetation type offers foraging opportunities when higher quality options are not available [29]. Additionally, the dense canopy cover offers better microclimatic conditions and cover from snow [29]. Weather conditions and the resulting need for shelter are therefore also an important driver that might cause deer to select habitats with lesser forage quality [30]. Other important abiotic factors are terrain height and spatial heterogeneity of the topography. While spatial heterogeneity of the topography allows for a varying plant phenology, actually extending the timespan of high quality forage availability [31], [29] have shown that terrain heights chosen by red deer maximize vegetation availability while avoiding agricultural and built-up land.

Another important set of factors is intra- and interspecific interactions. Increased deer density results in an increased use of less-preferable habitats [30]. Another key factor is the presence of predators such as large carnivores or human hunters. Naturally, large carnivores would have an important influence on habitat selection because they create a landscape of fear, where red deer would avoid certain habitat types and prefer habitats that offer a certain security [27, 32]. While large carnivores have recovered unexpectedly rapidly in some parts of Europe and even recolonize human-dominated landscapes [33], they are still mostly absent and even where they are returning, have mostly not established population sizes that will influence deer behaviour [27]. Instead, most deer populations experience a hunting regime, which creates a spatially and temporally varying landscape of fear, but different to the one that would be created by carnivores. For example, hunting laws restrict human hunting activity to certain timespans within a year and it is still common hunting practice to prefer stags for trophies rather than selecting the old, ill or otherwise weak as carnivores would do [34]. Furthermore, humans only hunt during certain times of the day and only in areas that are accessible. Therefore, red deer avoid the areas with high hunting pressure [27]. They also have been shown to occur more frequently with increased distance to settlements and roads so it seems they react negatively to the presence of all humans, not only hunters [29, 32].

In summary, former mining areas occur widely in modern landscapes and exemplify general land abandonment in areas where the former land use practices are not suitable or profitable anymore. As a key question will be how such abandoned landscapes that often consist of novel ecosystems will develop in the future, it is important to assess the role ecosystem engineers such as large herbivores might be able to play in order to maintain or promote a high biodiversity in them as well as affect various ecosystem services. The aim of our study was therefore to examine habitat preference of red deer at a former brown-coal mining area, which was not only shaped by the extraction of brown coal and the re-cultivating attempts afterwards, but is still managed by landowners and hunters to sustain high wildlife populations. This area consists also of novel ecosystems *sensu* Hobbs, with an anthropogenic topography, which is much more rugged than the naturally flat terrain in the area, a changed hydrology with a novel system of many small lakes, and forest plantations with a strong representation of exotic tree species such as *Pinus contorta*, many of which exhibit abundant regeneration and likely form self-maintaining populations. Furthermore, parts of the study site are essentially abandoned, since continued risk of subsidence makes it not safe to build or use heavy machinery. Other parts such as mowed grassy areas for deer feeding and agricultural fields still remain under heavy human influence. Based on the literature of red deer habitat selection in areas managed by hunters but currently lacking natural predators (although wolves *(Canis lupus)* are returning to Denmark), we expected the following factors to be important for habitat preference:

1. Red deer habitat selection is dependent on the availability of forage at a site. It was expected that red deer would prefer areas with high availability of forage to areas with low availability of forage. As red deer is a ruminant, foraging periodically during most of the day and also needing a huge amount of fodder input, this was considered the most important factor for habitat selection.
2. Red deer habitat selection is dependent on forest cover. Red deer prefer areas with high forest cover which are more suitable for hiding to areas with low forest cover. Even though natural predators are absent in the study area, the human hunting leaves the red deer sensitive for disturbances and we expected them to prefer areas with cover, especially for resting.
3. Red deer habitat selection is dependent on the distance to lakes that offer drinking water. Deer will prefer habitat patches in close proximity to water availability.
4. Red deer habitat selection is dependent on the terrain height. Deer might prefer higher elevations because they give a better overview of the surroundings and they are often areas with less human activity.
5. Red deer habitat selection is dependent on human activities (distance to buildings, roads and railways). Red deer will avoid humans and select the areas with least human disturbance.

## Material and methods

### Study area

**Fig 1:**
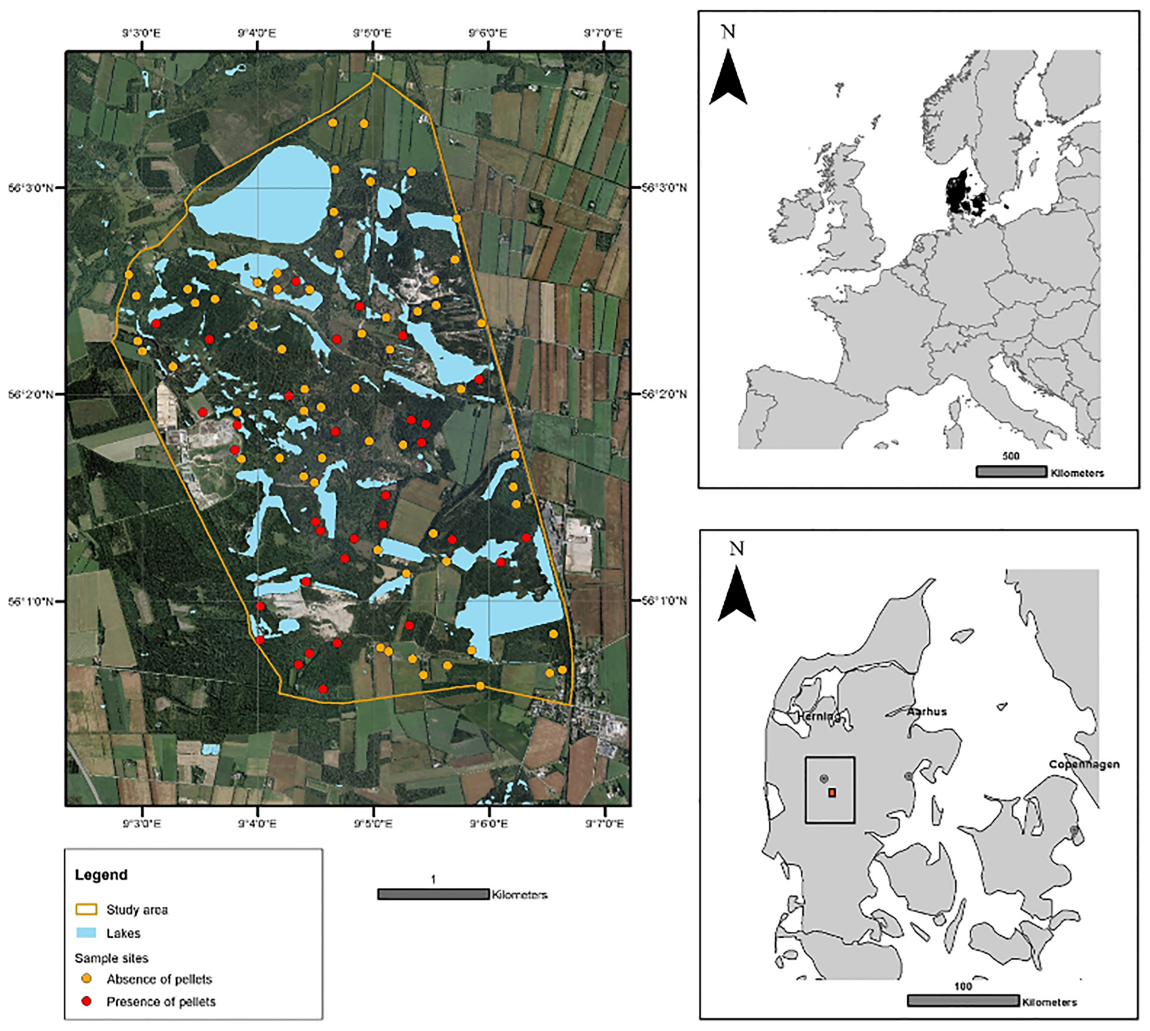
Study area, situated in the middle of Jutland (right bottom). 100 circular study sites with a 10 m diameter were randomly distributed over the whole area (left), red points depict findings of pellets in a study site while in orange dots, no pellets were found. Orthophoto printed under a CC BY license, with permission from COWI A/S, Denmark, original copyright 2014 (S1 Text) and ESRI basemaps printed under a CC BY license, with permission from ESRI and its licensors, original copyright 2014 (S2 Text).

The study was carried out in Central Jutland, Denmark, at a former brown-coal mining area in Søby (Fig 1). The study area covers 1,496 hectares and stretches from latitude 56°03’21N to 56°00’29N and from longitude 9°02’45E to 9°06’42E. Mean annual temperature is 7.5–7.6°C, with monthly temperatures from—3.5°C in the coldest month to 19.7°C in the warmest month, while mean annual precipitation ranges 802–813 mm. Situated in a flat glacial sandy outwash plain (sandur), up to 1971 the area was used for brown-coal mining and therefore now has a complex topography with many steep areas with still on-going changes, due to subsidence of areas up to several hectares wide. After the mining activities ceased afforestation was attempted, and the landscape nowadays consists of a patchwork of plantations, spontaneously established woodland, grassy areas, remnant heathland, little vegetated sandy areas, a few agricultural fields and a high density of smaller lakes. The vegetation includes abundant pines (the native *Pinus sylvestris*, but also the exotics *Pn*. *contorta* (most common tree in the area), *Pn*. *nigra* and *Pn*. *mugo*, intermixed with some spruce (*Picea abies*, *Pc*. *sitchensis*), European silver fir (*Abies alba*) and larch (*Larix spec*.). A number of broadleaved tree and shrub species are also common, notably the native *Betula pendula*, *B*. *pubescens*, *Sorbus aucuparia*, *Quercus robur*, *Fagus sylvatica*, *Populus tremula*, *Crataegus monogyna*, *Salix* spp. and *Sambucus nigra*, but also some exotics, notably *Prunus serotina* and *Quercus rubra*. Most of these species exhibit spontaneous regeneration. The understory contains graminoids, forbs, ferns, lichen, mosses, and small amounts of dwarf shrubs (*Calluna vulgaris*, *Empetrum nigrum*, *Vaccinium vitis-idaea*, *V*. *uliginosum*), but is often sparse in the conifer-dominated parts.

288 hectares of the study area are owned and managed by the Danish Nature Agency, while 1208 hectares are private land. In 2007 the whole area except the agricultural fields was set under a protection regime, which requires the open sand areas to be kept open for historical and cultural reasons, and that public access to the area is improved [35]. Because of the remaining danger of subsidence, there has so far not been any expansion of public access options and as a consequence, there are very few visitors. Hence, the location is relatively undisturbed except for periodic hunting and ongoing activities to promote the deer population, e.g. supplementary feeding and mowing of meadows.

### Sampling sites

The Danish Nature Agency (Naturstyrelsen) granted permission to carry out field work on the state-owned parts of the study area. As the largest part of our study area is privately owned, every land owner was asked to grant permission to do field work on his property beforehand. Data was collected 25^th^ of April to 10^th^ of July 2014. 100 sampling sites were randomly chosen with a minimum distance of 100 meters to reduce spatial autocorrelation among sites and ensure broad sampling of the study area (Fig 1). Lakes and buildings were excluded from the study area before placing the randomized sampling sites. Each site was located with the use of a Trimble Juno SB handheld GPS. When the site was located a sampling site of 10 meters in diameter was outlined with the use of 8 poles connected to one centre pole with strings (S1 Fig). In this way, the sampling site was divided into eight equally sized subdivisions to aid registration of vegetation and activity variable. If a sampling site was found to be on a road or in a swampy area, it was moved perpendicular to the road or lake border until the edge of the 10-meter circle would be just at the border of the road or water. 7 of the 100 samplings sites were excluded from the study because they were located inside a fenced agricultural field. Data collection was carried out with as little disturbance of the deer population as possible.

### Red deer activity

Denmark’s red deer population was estimated approx. 24.000 in 2015 with a rising tendency. In the region Central Jutland in which our study area is situated, red deer population was estimated approx. 3.600 in 2015, with a hind-calf ratio of 1,5:1 [36]. The study area and the neighbouring plantations (353 hectares) are part of a bigger area which local hunters estimate to hold approximately 500 red deer, 100 fallow deer (*Dama dama*), and 100 roe deer (*Capreolus capreolus*) (M. Hauge, pers. commu.). This would result in population densities of 27 red deer/km^2^, 5 fallow deer/km^2^, and 5 roe deer/km^2^. As home ranges of red deer in Danish landscapes are large (50 km^2^ for female red deer and even larger for males [37]), the local population is also using the adjacent landscapes outside the study area. As red deer is now common in this part of Denmark, the local population is interacting with other populations on a regular basis. This study focuses on the activity of red deer as it is the largest deer with the highest population numbers in the area and therefore expected to have the largest ecological impact. Furthermore, we were especially interested in red deer as a species that only recently started spreading again on the European continent.

Faecal pellets from red deer were used as an indicator of relative use of the sampling site (S2 Fig). Faecal pellet count is a method widely applied in studies of ungulate habitat selection [32, 38, 39] and has been shown to be a reliable method by comparison to radio telemetry and GPS collar data [40, 41]. The faecal pellets were identified to be from red deer, fallow deer or roe deer following [42]. If a faecal pellet group was located more than 30 cm away from another faecal pellet group, it was counted as a separate case. As red deer are social animals with herd behavior, presence of faecal pellets (1) or absence of faecal pellets (0) was chosen as the dependent variable to reduce stochastic variability in pellet findings.

To validate the use of pellet counts as an activity measure, red deer activity was also examined with Spypoint BF-6 Invisible LEDs camera traps, put up to survey 30 sampling sites. These were randomly chosen from the 100 sampling sites, separated by at least 150 m to reduce spatial autocorrelation and ensure broad sampling of the study area. Each sampling site was located with a handheld GPS and the camera was set in a nearby tree so that it would film the sampling site area. The cameras were placed in a 2–3 weeks period to record deer activity at each sampling site. Each camera was set to film 10 seconds whenever a movement was registered by the sensor and thereafter have a delay of 15 minutes where it would be insensitive to movement.

Bark stripping (number of trees with wounds) was noted as another indicator of relative use of the sampling site. As this measurement could only be recorded for sampling sites with trees, it was not used as a dependent variable in the habitat selection analysis.

### Vegetation sampling

To provide a measure of food availability in each sampling site the amount (percentage cover) of understory vegetation, moss, lichen and bare ground was noted in all eight subdivisions per site. Cover was evaluated in a logarithmic scale with the intervals: ≤12.5%, >12.5%–25%, >25%–50% and >50% (S1 Table). The average of all subdivisions was estimated for each category. “Understorey vegetation” (UV) was all vegetation that was not moss or lichen and situated from ground level up to 1 m above ground, e.g. herbs, grass, smaller trees and leaves from bigger trees located below 1 m height. “Bare ground” (BG) was all non-vegetated floor areas as well as ground covered with fallen dead tree trunks or occupied by living tree trunks, representing areas providing no forage for deer.

To measure vegetation density, the amount (%) of each subdivision which would be considered inaccessible to a red deer (e.g. if the shrub layer was to dense to allow walking through or if there was dead wood laying around that should prohibit red deer passing over it) was noted also in a logarithmic scale with the intervals: 0–12%, 12%-25%, 25%-50% and 50%-100% and termed “understorey density” (UD) (S1 Table). The average of all subdivisions was calculated by dividing the sum of the median of each interval with the total number (8) of all subdivisions. Tree cover (TC) was evaluated in each subdivision, by standing in the middle of the sampling site and using an inclinometer to make sure to look up in a 90° angle, if leaves were seen there was cover, if sky was seen no cover. Tree cover was noted as amount of subdivisions with cover divided by the total number (8) of subdivisions.

### Estimation of habitat variables

Habitat selection in ungulates is affected by human features in the landscape like roads, railways and buildings [43]. As drinking places are important to red deer in the summer [44], distance to lakes was also included in the analysis. With the use of Geoinformation Systems (GIS) the shortest distances from sampling sites to the following features were estimated: buildings, roads >6 m wide, roads 3m-6m wide, roads <3 m in width, railways, and lakes [45]. All GIS operations were performed in ArcGIS 10.1.

**Fig 2:**
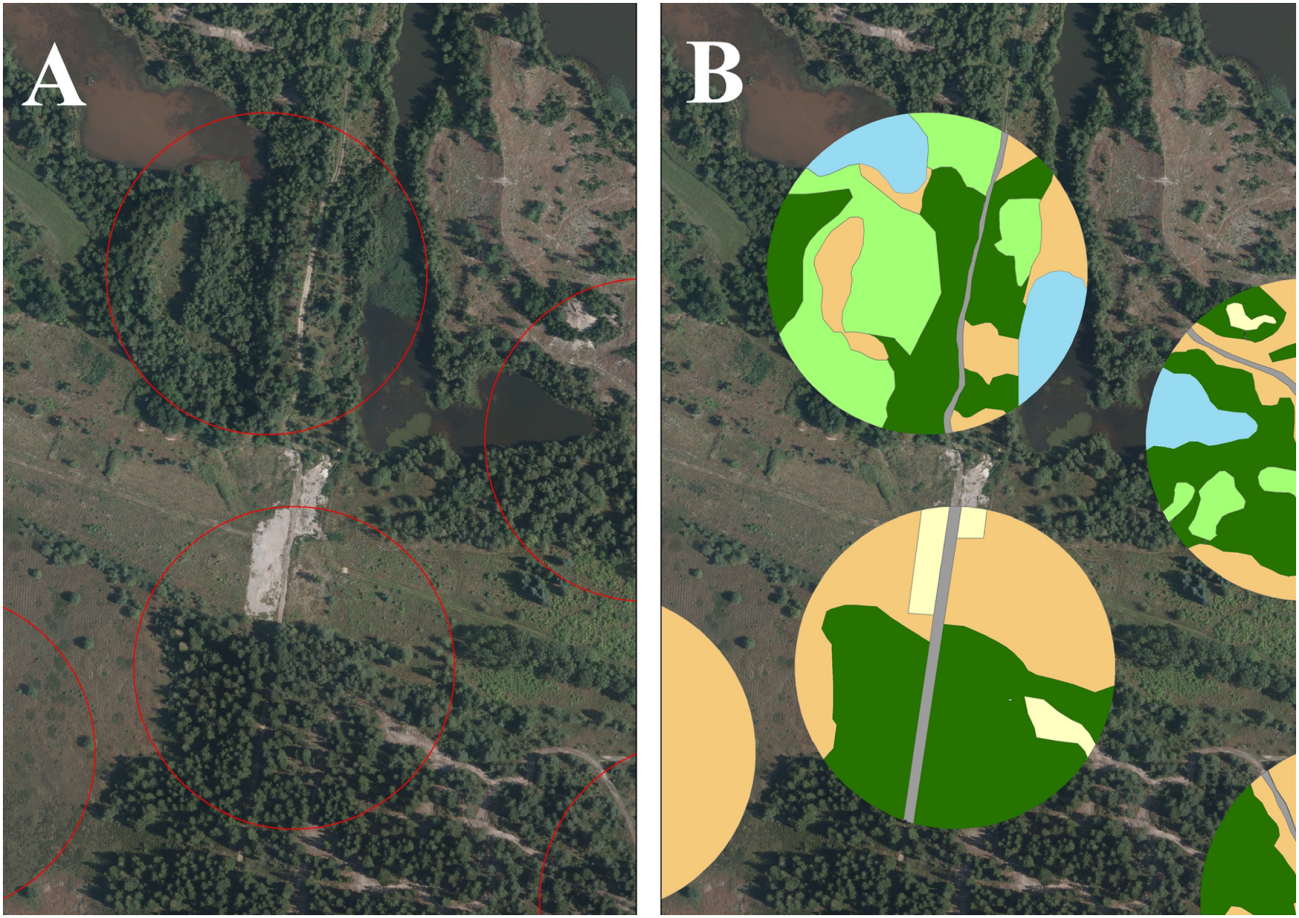
Example of digitalization of patches of landscape types inside sampling site buffer zones of 100 m radius. A shows the orthophoto with the buffer zone (red circle) before estimation of the features, and B shows the same buffer zones afterwards (dark green = needle forest, bright green = deciduous forest, blue = lakes, brown = open land, yellow = sand). Orthophoto: 16 cm resolution, printed under a CC BY license, with permission from COWI A/S, Denmark, original copyright 2014 (S1 Text).

The habitat around each sampling site was characterized by a 100 m in radius circular buffer zone (Fig 2). In each buffer zone the following features were estimated by interpretation and digitalization of aerial photographs and from observations of forest type in the field [45]: deciduous forest (A_DECI), coniferous forest (A-CONI), open areas, sandy areas, lakes, buildings, roads (Fig 2). For each feature the surface area was estimated, thereby giving the relative amounts of each habitat type theoretically available to the red deer walking through the sampling site.

As terrain variation within the study site is relatively high due to the former mining activities, its importance on habitat selection of red deer was also examined. It should be noted that terrain variation might be related to other habitat characteristics important to red deer such as a lower human disturbance regime because there are e.g. no houses built on areas with former mining activity. It is thus likely that areas with high terrain variation or high mean terrain height might be primarily selected not for their terrain characteristics but for other habitat characteristics red deer find favourable there. In ArcMap a digital terrain model [46] was used to calculate maximum and mean terrain height, and the standard deviation of terrain inside the buffer zone. As tree heights and therefore stand age could also influence red deer habitat selection, tree heights in the study area were derived from a digital object model, DOM. This DOM was created by subtracting the digital surface model, DSM [47] from the digital terrain model, DTM [46]. For each buffer zone, maximum tree height, mean tree height and standard deviation of tree heights was thereafter calculated as maximum DOM heights, mean DOM heights and standard deviation of DOM heights in ArcMap.

### Statistical analysis

The activity measurements recorded with wildlife cameras were used to evaluate the accuracy of pellet sampling as a measure of red deer use of the sampling sites. Because of wind disturbance, which activated the cameras in some places and not in others, the amount of time the cameras recorded differed (7.36–17.96 days, mean = 14.14 days, SD = 3.49). Deer frequency at the given camera site was therefore measured as the number of red deer recorded by the camera divided by the amount of days the camera had recorded (number of deer/amount of days). The camera-estimated frequency was then compared between sampling sites with (1) and without (0) pellets. Data were analysed with an analysis of variance (ANOVA) in the statistical program R [48].

The interrelationships among the many predictor variables (S2 Table) were assessed using Principal Component Analysis (PCA) performed in JMP 10.0.2. The five main principal components accounted for 57% of the variation in the predictor variables, and only the six predictor variables with the highest loadings were considered in the subsequent modelling: DTM_MEAN (terrain mean height (m) in 100-meter radius buffer zone) and A_CONI (area (m^2^) covered with coniferous forest inside 100-meter radius buffer zone) both loaded strongly on PC1, which describes a gradient from low cover of conifer trees on flat terrain to high conifer cover on complex terrain, reflecting conifer planting and spontaneous colonization of former dig sites. By contrast, deciduous trees are more abundant in the flatter parts of the study area. TC (percentage tree cover at the sampling site) and UV (percentage understory vegetation cover) both loaded strongly on PC2, in opposite directions, describing a gradient from open areas with dense ground vegetation to closed tree stands with little undergrowth. DTM_MEAN also loaded strongly on PC3 together with TC and UV, reflecting an association of low tree cover and dense ground vegetation with low terrain height. D_BUILD (distance to buildings) loaded strongly on PC4. A_DECI (deciduous forest cover) loaded strongly on PC5. The selected variables were checked for collinearity (Pearson r < 0.50, except UV vs. TC with r = -0.61).

Multiple binary logistic regression was used to estimate the influence of the selected six predictor variables on red deer habitat selection. Different configurations of adding and removing predictor variables were tested and the best model was selected by comparing the Akaike Information Criterion (AIC) value, selecting the model with the lowest AIC. The logistic regression modelling was carried out in JMP 10.0.2.

## Results

**Fig 3:**
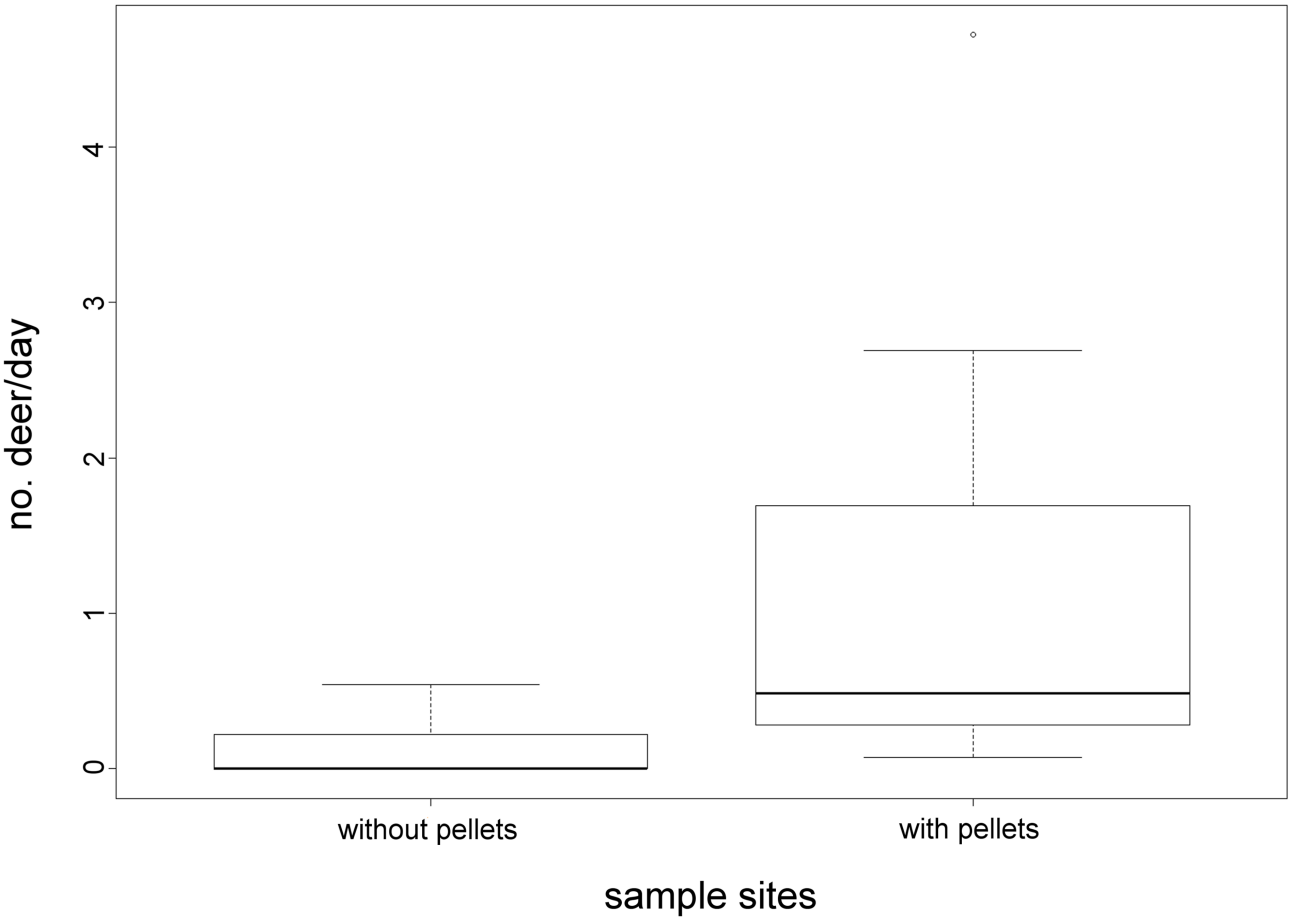
Deer frequency at camera sites. Measured as the number of red deer recorded divided by the amount of days the camera recorded (no. deer/day) in sampling sites with or without pellets. A t-test showed a significant difference (p = 0.00351) between the camera recorded activity in sampling sites with no pellets and camera recorded activity in sampling sites with pellets.

Faecal pellets from red deer occurred in 19 out of 93 sampling sites (Fig 1), and the camera trap data indicated much higher red deer activity at sites with faecal presence (Fig 3).

**Fig 4:**
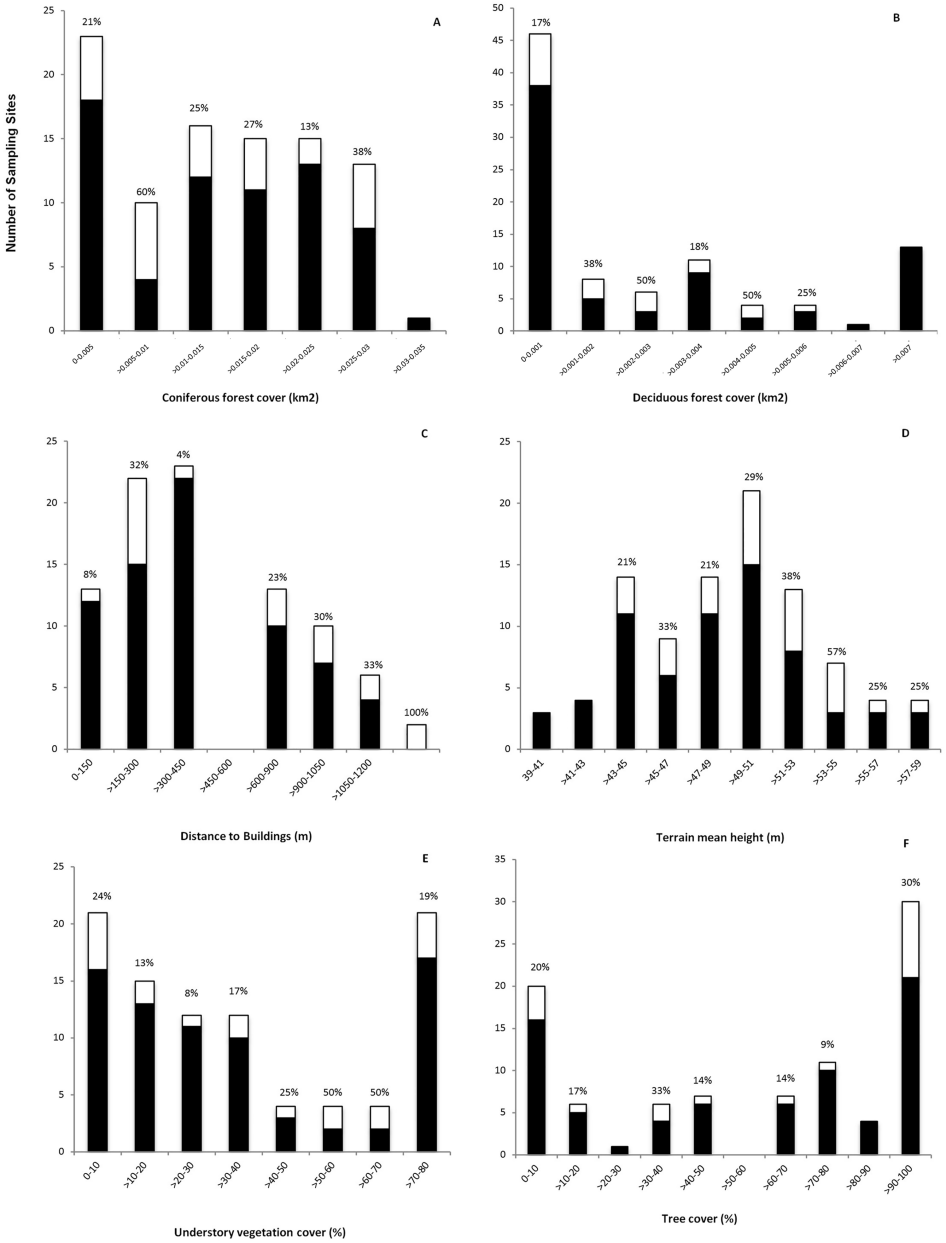
Distribution of sample sites with pellet counts (indicating red deer presence) and without pellet counts (indicating red deer absence) across different categories of the six selected predictor variables. The bars show the number of sites observed in each category. The white end of each bar shows the proportion of sampled sites with presence of red deer pellets while the black parts depict the proportion of sampled sites with absence of red deer presence. Above each bar the percent of sites in that interval with red deer pellets is shown.

The best logistic regression model included all six variables that were identified in the PCA (AIC = 84.7, the next best model excluding A_CONI had AIC = 87.2). The model showed that red deer preferentially used sample sites with high tree cover (TC), but also with high understory vegetation cover (UV), greater distance to buildings (D_BUILD) and higher mean terrain height (DTM_MEAN). On the other hand, high deciduous forest cover (A_DECI) and high coniferous forest cover within a 100m buffer around a sample site (A_CONI) were negatively correlated to red deer habitat selection (Table 1, Fig 4).

**Table 1:** Logistic regression analysis.

Independent variable	Coefficient	Chi-squared*	p-value
**A_CONI**	-0.0000915	4.31	0.0376
**A_DECI**	-0.000284	4.32	0.0380
**D_BUILD**	0.00471	9.11	0.0025
**DTM_MEAN**	0.280	8.25	0.0041
**UV**	0.0497	5.43	0.0198
**TC**	2.923	4.96	0.0260
**Model Chi Square**	24.8		
**AlC**^**c**^	84.7		
**Generalized R**^**2**^ **p—value**	0.367 0.004		

The model statistic shows the six independent variables that were selected to explain habitat selection by red deer. All selected variables show a significant influence (p-value < 0.05) on the habitat selection.

* Shows Wald tests for the hypotheses that each of the parameters is zero.

**Fig 5:**
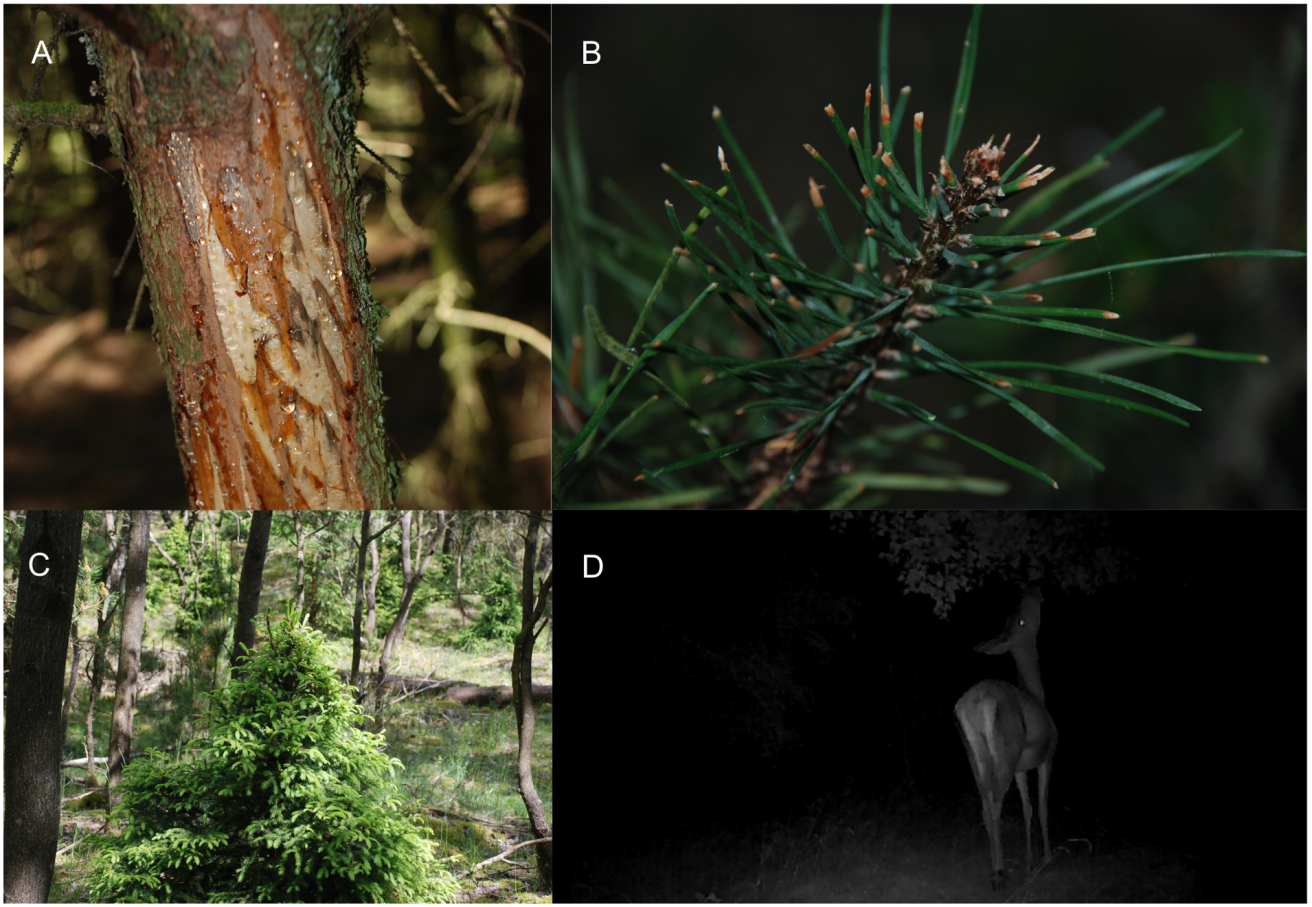
Examples of red deer browsing impact in the study area. Bark stripping (A) and browsing of buds and shoots (B), inducing an abnormal and compact growth of conifers induced by browsing (C). Still picture from a wildlife camera recording of a red deer hind feeding from a *Betula spec*. tree in the night (D).

Our field observations showed that red deer affected trees in various ways (Fig 5). We observed trees with severe bark stripping, especially in large *Picea abies*. At our site we observed browse lines around 160 cm, while small conifers under this height were observed with a very dense structural growth, with indications of recent browsing.

Red deer activity was highly spatially heterogeneous: High red deer activity can be found mostly in the southern parts where the mining activity had been most intense, resulting in more complex terrain, making human access more difficult. At the same time red deer activity was seldom recorded in the northern parts, where terrain heights are generally lower and human activities like buildings, roads, railway and agricultural land are more often situated.

## Discussion

Our study results support hypothesis one, that red deer habitat selection is dependent on the availability of forage at a site; hypothesis two, that red deer habitat selection is dependent on forest cover; hypothesis four, that red deer habitat selection is dependent on the terrain height and hypothesis five; that red deer habitat selection is dependent on human activities. The results demonstrate a strong preference in red deer for the more remote parts in the study area, highlighting the importance of human activities in shaping habitat selection in novel ecosystems in this large herbivore. The study also reveals that habitat selection is influenced by vegetation characteristics and topography, with red deer preferentially occurring in habitats with a high cover of understory vegetation. They also preferred higher percentage of tree cover at sampling sites whereas high deciduous or coniferous cover within the 100m buffer around the sample sites decreased deer habitat preference. Furthermore, they preferred higher mean terrain heights. Our findings do not support hypothesis three, that red deer would select areas closer to drinking water which can probably be explained by the high abundance of large and small lakes in the former mining area, so deer can probably access water from any given part of the study area during their daily activity pattern.

### Vegetation characteristics influencing habitat use by red deer

Deer in our study were shown to prefer high ground plant cover directly at the sample site but they were also shown to prefer sample sites with high tree cover. Deer apparently preferred sites with high fodder availability but they also seem to appreciate high tree cover. So they might prefer forest types with an abundant understory vegetation, where they can forage and find cover at the same time. This was also shown in a study within non-native lowland pine forests in Hungary, where red deer habitat selection was related to understory characteristics instead of forest type [49]. On the other hand, they might also use different vegetation types for different purposes: open areas with low tree cover and high understory vegetation cover for feeding and forest with high tree cover during resting periods (Fig 4F), varying their preference for certain vegetation characteristics depending on certain activities during the day. From our field observations, sample sites in forests often consisted of little understorey vegetation, probably being rather used for transit or resting. The finding that red deer preferred sample sites with low deciduous/coniferous forest cover in the 100m buffer zones might rather represent a preference for openings within the closed forest cover that have been shown to be favoured feeding sites [27]. It has been shown before that deer need resources present in different habitat types for different activities, resulting in smaller home range sizes found in heterogeneous landscapes [28]. The former brown-coal mining area at Søby shows high heterogeneity in tree and understory vegetation, with small patches of different vegetation types being found within relatively small distances from each other and being easily reached within the normal daily activity pattern of red deer [50].

Overall, red deer seem to benefit from the heterogeneous landscapes and the typical patchy vegetation patterns of novel ecosystems that emerge after industrial land use sites are abandoned. Especially mining activity leaves landscapes where remnant forest patches might be interspersed with early successional stages that provide more open habitat patches with high forage abundance, while the forest patches offer sufficient cover. Re-cultivation measurements often also include afforestation of parts of the areas (as was done in Søby e.g. by *Pinus contorta* plantations) with fast growing tree species that are adapted to the abiotic conditions [51]. If no socio-economic interests in such areas remain, abandoned industrial sites and their novel ecosystems provide red deer habitat areas that can easily be recolonized and might present stepping stone habitats in densely populated European landscapes where natural habitats often became fragmented [25].

### Topography effects on habitat use by red deer

Habitats were more often selected in higher parts of the terrain in the study area than in lower parts. Areas positioned at high altitude might provide a better overview of the area and red deer might also be better able to smell, hear or see approaching humans from higher terrain. But it is also likely that the correlation between habitat use and mean terrain height is affected by other factors correlating with mean terrain height, such as human disturbance: Buildings, roads, railway and agricultural land are often situated at lower terrain heights in easily accessible areas resulting in high human disturbance regimes there. Additionally, higher terrain might be more challenging to traverse by humans, resulting in lower human activity. In some cases, high quality forages are situated at high altitudes and might be the more important reasons why red deer activity was recorded there. The choice for a certain terrain height by red deer has been attributed to food resources, climatic condition and human disturbance before, with deer apparently keeping to certain altitudes to avoid human activity but low enough to ensure good forage availability [29].

With these findings one could argue that high mean terrain height is not a necessary habitat attribute required by red deer per se. Instead, increased terrain height, slope and ruggedness might promote lower human disturbances. Especially in the absence of large predators, these characteristics will render landscapes favourable to deer as long as other habitat characteristics such as forage availability are still suitable. In densely populated European landscapes, abandoned post-industrial sites, where terrain alterations or other legacies keep human disturbance low, might be especially suitable as large wild herbivore habitat. There are examples of a successful establishment of large herbivore habitats at former industrial sites with little human influence e.g. a nuclear weapons manufacturing complex in Colorado where bison *(Bos bison)* can be found free-roaming today [52] or a former coal mine in the Alberta Rockies which was restored as suitable Rocky Mountain bighorn sheep *(Ovis canadensis canadensis)* habitat [53].

### Human activity restricting habitat use by red deer

Habitats were selected more frequently by red deer when distance to buildings increased. This is consistent with findings of preceding studies of red deer habitat selection under anthropogenic influence [50, 54, 55]. In Denmark with the recent historical lack of predators large enough to take down red deer, the only threat comes from hunting and encounters with motor vehicles. In a study by [50] the number of red deer killed in Denmark by hunters significantly decreased with increasing building density in the surrounding area. This could be due both, deer avoiding human settlements, and laws prohibiting hunting near human settlements. If disturbed by human activities, including tourists, red deer appear to shift foraging activity to forest and away from open areas [54, 56].

Human disturbance does therefore determine where red deer will be found and where not. If red deer avoid certain areas due to permanent (e.g. settlements) or periodical (e.g. hunting) human presence this has of course also implications for the influence of red deer on these areas: Deer browsing will probably be reduced, allowing vegetation succession while other parts of a landscape with less disturbance will face higher deer activity and therefore higher grazing pressure. Humans produce a completely new ‘landscape of fear’ that functions differently from the one induced by other predators. It was shown for roe deer that predation risk by humans was higher in open habitats whereas predation risk by lynx was higher in forest habitats [57]. As also stated by [56] deer shifts activity away from open areas and into forests if presented with human activities and this is probably especially true if natural predators that could pose a risk in the forests are absent from the landscape. Both, human and carnivore predators do influence the way red deer use a landscape and both create spatial heterogeneity with areas that face different grazing pressure depending on the red deer activity. But as human hunting activity is more easily manageable than predation by carnivores and often the only threat where carnivores are absent, a management plan of where and how to hunt might actually help in determining which areas of a landscape are used more frequently by deer and which are not–and might therefore be a valuable conservation tool for establishing heterogeneous landscapes.

### Landscape management by hunters and landowners

The plantations in Søby and the area in general are mainly managed with the goal of improving hunting experience and to a much lesser extent for forestry. Red deer are fed with beets or grain during autumn and wintertime in most areas except for the areas owned by The Danish Nature Agency, in which it is prohibited (H. Jensen, personal communication). During the summer, some landowners manage grassy areas for supplementary forage. During data collection, the study site contained a high abundance of natural forage, but red deer were often observed foraging in the managed meadows. These high-quality forage grounds likely attract deer to these areas and explain high red deer activity recorded in sample sites close by. Supplementary feeding and habitat configuration strongly affect the spatial ecology of red deer. [58] found the smallest home range size of red deer populations in areas with supplementary feeding, maintaining the same home range size year-round. Supplementary feeding may maintain the deer at an unusually high density and it is doubtful whether this activity helps to protect crops, forest or other natural vegetation because possible positive effects are often undermined by the induced population growth [59].

Most common management practices such as supplementary feeding lead to high population densities which might cause problems for the deer population, e.g. enhancing spread of disease [60]. Animals might also start relying on supplementary feeding, raising the question how ‘wild’ such populations truly are [61]. On the other hand, little is known on the ‘natural’ population density of red deer, as foresters often try to keep population densities in forests where they occur low in order to prevent damage on trees [62]. In order for red deer to truly function as ecosystem engineer and help maintaining open habitats, which are currently often endangered by forest succession and only insufficiently managed by livestock grazing [63], population densities might be allowed to rise in some areas, e.g. post-industrial sites with potential high nature conservation value, where tree damage and interruption of succession is desired.

### Influence of the red deer on the landscape

The red deer affected trees in various ways in our study area, but mainly through bark stripping. Bark stripping and rubbing antlers, to shed the velvet, can cause lethal damage to young trees [64], and different tree species will show different relationships between woody plant growth (fitness) and herbivore browsing severity [21, 65]. Bark wounds can leave trees more vulnerable to fungi [66]. We also observed high levels of browsing which often results in growth of lateral shoots and development of a more bushy structure [22].

These observations suggest that the deer are slowing the growth of both conifers and deciduous canopy by producing shrubby growth forms where early age classes of trees are found, and a browse line where trees were already taller than the deer. In the long term, and in the absence of management interventions, this should result in shrubby, dense formations in early successional areas and open understories at the edges of and interior to more mature forests. In general, this may arrest or slow successional processes in the deer’s preferred habitat areas. Deer have been shown to damage tree saplings and reduce survival rates and heights of older trees [67]. Large population densities of ungulates have been shown to cause severe damage [68] and even low population densities of ungulates can have significant impacts on forest regeneration [69]. However, bark stripping, browsing and fraying have been shown to be very selective and their impact varies for different tree species [70]. Thus, even at a relatively high population density, the effects of red deer habitat preferences on forest dynamics will be highly patchy and heterogeneous, allowing regeneration to occur in parts and contributing to high spatial variation in vegetation structure and composition. Understory plant richness in boreal forests was shown to be enhanced with larger red deer population densities [71] indicating their potential as possible tool to enhance biodiversity of forest understories.

## Conclusion

We found that red deer prefer parts of the study area with high forage abundance, high forest cover, and low human disturbance, which was probably also why high terrain was preferred. Even though their use of the area is spatially heterogeneous and the areas they use often determined by the human activities in the area, they were able to recolonize this novel ecosystem quite successfully. Semi-natural open habitats are often prioritized for nature conservation, but their current management e.g. by livestock grazing was shown to often be insufficient in order to keep them from being encroached by shrubs and trees [63]. Thus, wild herbivores such as red deer could be potential keystone species for maintaining semi-open vegetation in post-industrial, but also remnant natural areas in Europe [24]. They are not only able to slow down or prohibit forest regeneration by browsing and grazing, but also might be valuable seed dispersers helping to connect habitats in patchy landscapes and therefore enabling short and long distance plant dispersal [72–75].

Persistent human activities, such as recreation or hunting, may lead to higher heterogeneity in deer herbivory effects at the landscape scale. The ability of red deer to find areas to avoid human activities in turn is positively affected by the topographical heterogeneity and instability typical of an abandoned and spontaneously recovering post-industrial site. The positive and negative forms of interaction, including improving food and cover availability, and hunting and disturbing the deer, are likely to influence the trade-offs that deer make when deciding what and where to eat. More complex than the “landscape of fear,” the “landscape of human interaction” may be highly context-dependent across abandoned agricultural and industrial sites. In this sense, red deer can be distinguished from domestic species such as cattle, horses, or sheep, which can also be used to maintain open woodlands, but which often need to be managed deliberately for heterogeneity in time and space [76]. Management of novel ecosystems with the use of red deer as a means to achieve desired vegetation patterns and heterogeneous landscapes could probably benefit from an “open-ended” project approach [77], where the interaction between human management, land use, red deer herbivory and vegetation development are carefully monitored and the management of red deer populations is adapted accordingly in order to achieve nature and landscape conservation goals.

## Supporting information

S1 Fig Illustration of the sampling site. Graphic illustration by Thorlak Solberg (left) and photo of sampling site (right).
(TIF)

S2 Fig Different shapes and variations of red deer droppings. (TIF)

S1 Table Examples of assessment of vegetation percentage cover. Understory vegetation (UV), moss (Moss), bare ground (BG) and percentage density were evaluated in each subdivision of a sample site.
(DOCX)

S2 Table The independent variables included into the statistical analysis of habitat selection. There are five different types of variables: digitalized areas of different land covers within a 100-m radius buffer zone around each plot (area of one buffer zone = 31,350 m^2^), distances mapped from sampling site centre to the nearest feature, terrain height and tree height within the 100m-buffer zone and the vegetation cover data sampled in the field as percentages of the total area of a sampling site.
(DOCX)

S1 Text Copyright permission Figs 1 and 2. Granted copyright permission by COWI.
(DOCX)

S2 Text Copyright permission Fig 1. Granted copyright permission by ESRI.
(DOCX)

S1 File Complete original data. File containing all data derived by fieldwork.
(XLSX)
