# Supplementary figures and images for: Large herbivores in novel ecosystems - Habitat selection by red deer (Cervus elaphus) in a former brown-coal mining area
Source: PLoS One. 2017 May 15;12(5):e0177431. doi: 10.1371/journal.pone.0177431 (PMC5432106; doi:10.1371/journal.pone.0177431)

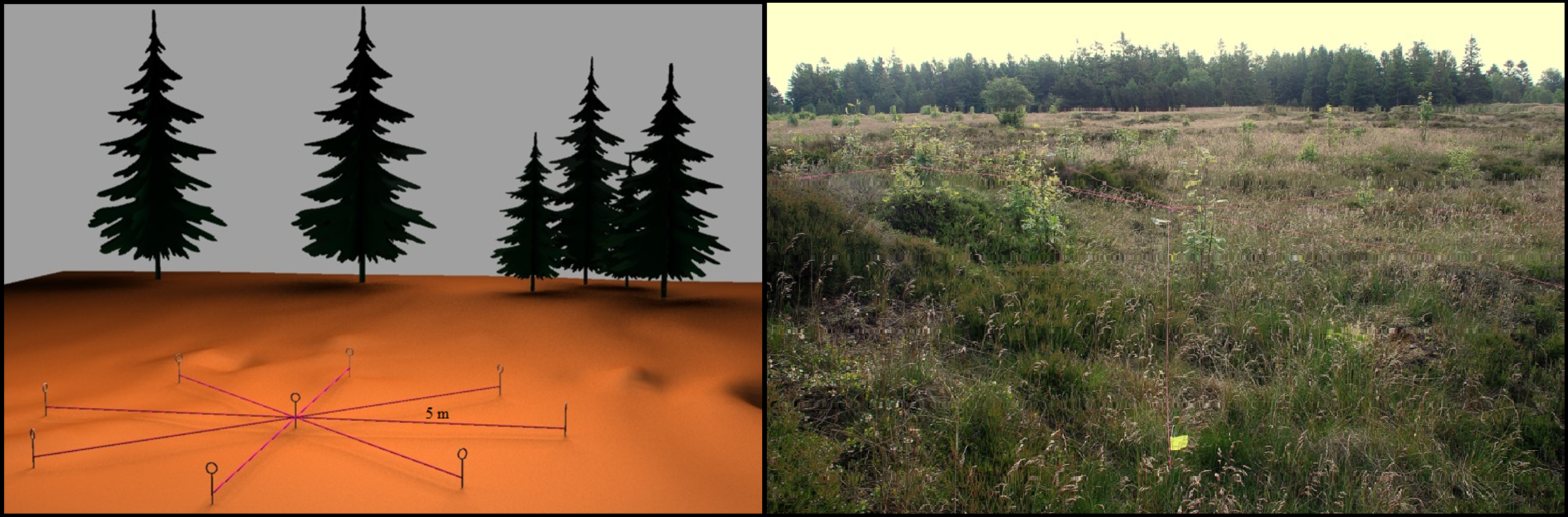

Supplement: S1 Fig — Graphic illustration by Thorlak Solberg (left) and photo of sampling site (right). (TIF) [file pone.0177431.s001.tif]

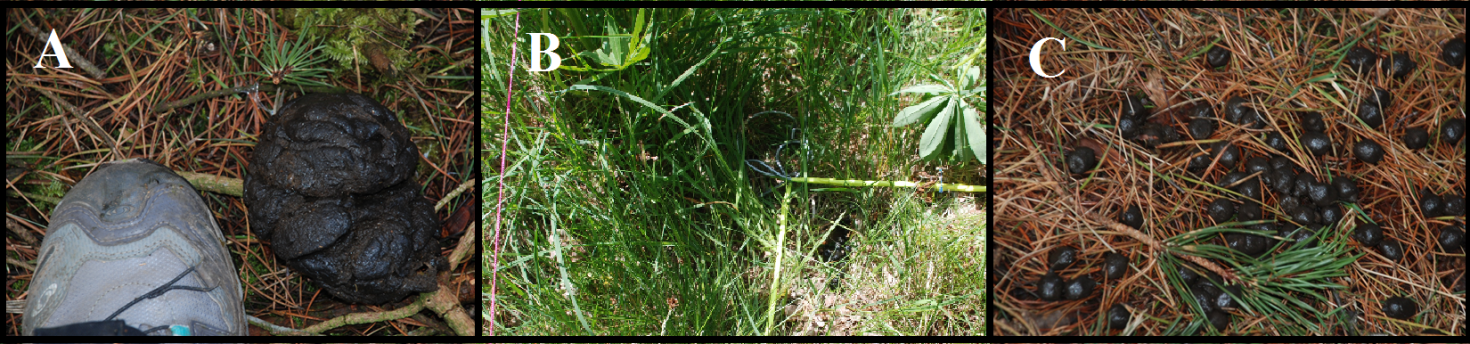

Supplement: S2 Fig — (TIF) [file pone.0177431.s002.tif]
