# Supplementary material for: Large herbivores in novel ecosystems - Habitat selection by red deer (Cervus elaphus) in a former brown-coal mining area
Source: PLoS One. 2017 May 15;12(5):e0177431. doi: 10.1371/journal.pone.0177431 (PMC5432106; doi:10.1371/journal.pone.0177431)

S1 Table Examples of assessment of vegetation percentage cover. Understory vegetation (UV), moss (Moss), bare ground (BG) and understory density were evaluated in each subdivision of a sample site.

**Understory vegetation**

####
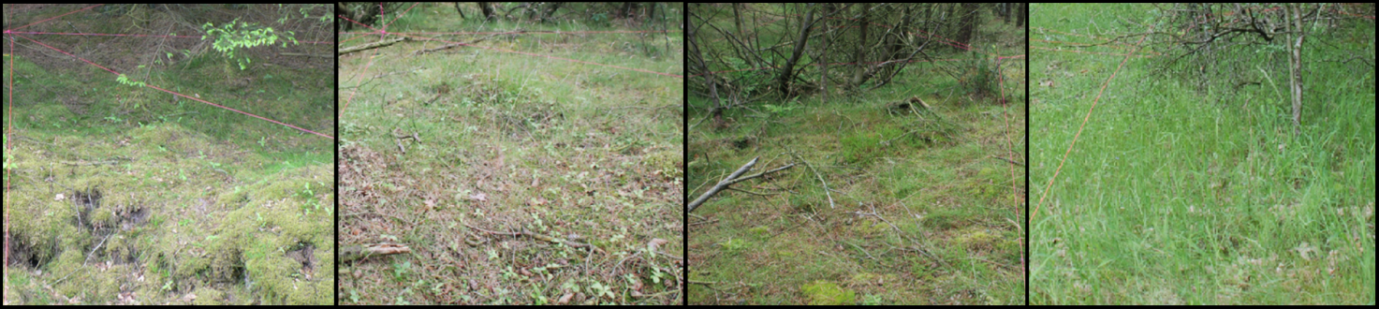

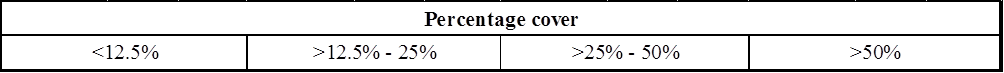


**Moss**

####
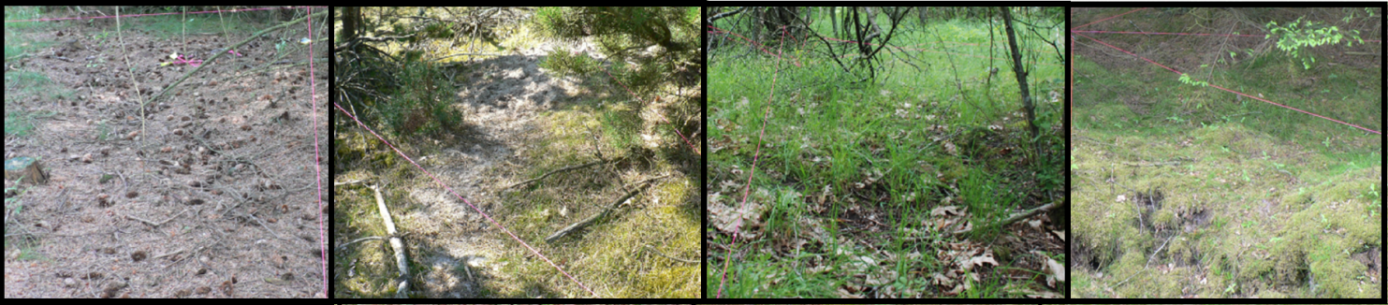


#### Bare ground

####
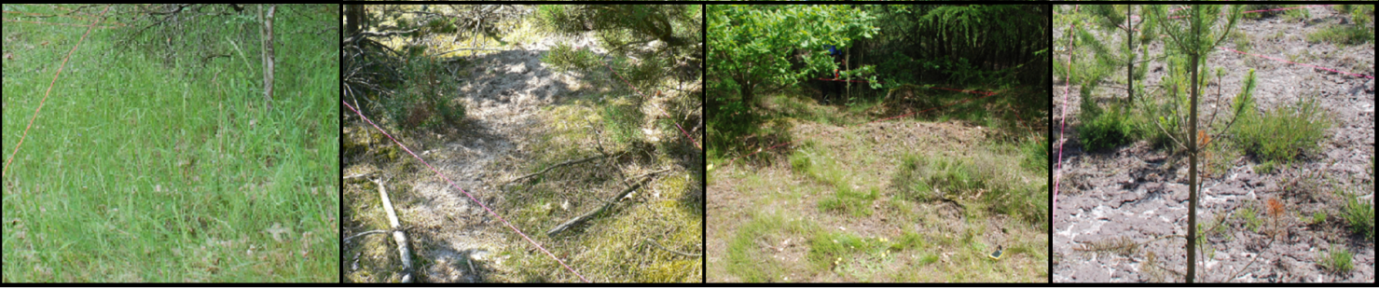


#### Understory density

####
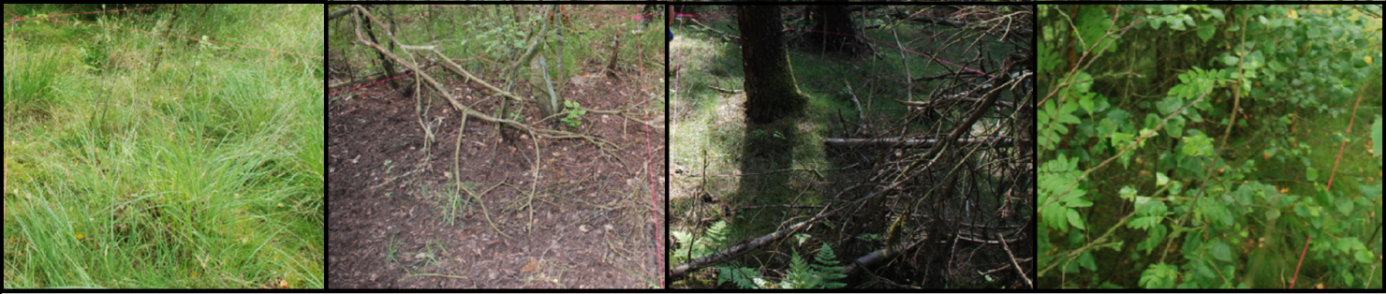

Supplement: S1 Table — Understory vegetation (UV), moss (Moss), bare ground (BG) and percentage density were evaluated in each subdivision of a sample site. (DOCX) [file pone.0177431.s003.docx]
