# Supplementary material for: Large herbivores in novel ecosystems - Habitat selection by red deer (Cervus elaphus) in a former brown-coal mining area
Source: PLoS One. 2017 May 15;12(5):e0177431. doi: 10.1371/journal.pone.0177431 (PMC5432106; doi:10.1371/journal.pone.0177431)
Supplement: S2 Table — There are five different types of variables: digitalized areas of different land covers within a 100-m radius buffer zone around each plot (area of one buffer zone = 31,350 m2), distances mapped from sampling site centre to the nearest feature, terrain height and tree height within the 100m-buffer zone and the vegetation cover data sampled in the field as percentages of the total area of a sampling site. (DOCX) [file pone.0177431.s004.docx]

S2 Table. The independent variables included into the statistical analysis of habitat selection. There are five different types of variables: digitalized areas of different land covers within a 100-m radius buffer zone around each plot (area of one buffer zone = 31,350 m^2^), distances mapped from sampling site centre to the nearest feature, terrain height and tree height within the 100m-buffer zone and the vegetation cover data sampled in the field as percentages of the total area of a sampling site.

| Digitalized | Unit | Minimum area | Mean area | Maximum area | Description |
| --- | --- | --- | --- | --- | --- |
| A_DECI | m^2^ | 0 | 3359 | 25880 | Deciduous forest |
| A_CONI | m^2^ | 0 | 13757 | 31350 | Coniferous forest |
| A_FOREST | m^2^ | 133 | 17117 | 31350 | Deciduous + Coniferous forest |
| A_OPEN | m^2^ | 0 | 7195 | 28059 | Open land (meadow, heath, grass field) |
| A_LAKE | m^2^ | 0 | 9793 | 28059 | Lakes |
| A_SAND | m^2^ | 0 | 646 | 14468 | Sand with only sparse vegetation |
| A_AGRI | m^2^ | 0 | 2598 | 27979 | Agricultural fields |
| A_AGRI_F | m^2^ | 0 | 245 | 12788 | Agricultural fields with electric fencing to keep out red deer |
| A_ROAD | m^2^ | 0 | 505 | 3822 | All types of roads |
| A_RAIL | m^2^ | 0 | 43.5 | 1644 | Railway |
| A_BUILD | m^2^ | 0 | 219 | 7803 | Buildings (houses, industry) |
| Distances | Unit | Minimum length | Mean  length | Maximum  length | Description |
| D_LAKE | m | 4 | 111 | 435 | Lakes |
| D_RAIL | m | 10 | 1425 | 3065 | Railway |
| D_ROAD_3_6 | m | 15 | 519 | 1367 | Roads from 3 - 6 meters wide |
| D_ROAD_6 | m | 6 | 1811 | 2967 | Roads > 6 meters wide |
| D_TRANS | m | 6 | 430 | 1304 | Railway + Roads 3-6 m + Roads > 6 m |
| D_ROAD_DIRT | m | 1 | 166 | 627 | Small dirt roads |
| D_BUILD | m | 27 | 400 | 1132 | Buildings (houses, industry) |
| Terrain height | Unit | Minimum height | Mean  height | Maximum  height | Description |
| DTM_STD | m | 0.139 | 2.03 | 8.27 | Standard deviation of terrain height |
| DTM_MEAN | m | 39.6 | 48.8 | 58.5 | Mean height of terrain |
| DTM_MAX | m | 42.7 | 54.5 | 75.8 | Maximum height of terrain |
| Tree height | Unit | Minimum  height | Mean  height | Maximum  height | Description |
| DOM_STD | m | 1.06 | 3.45 | 6.86 | Standard deviation of tree height |
| DOM_MEAN | m | 0.283 | 5.3 | 16.5 | Mean tree height |
| DOM_MAX | m | 7.01 | 13.6 | 23.5 | Maximum tree height |
| Field data | Unit | Minimum  percentage | Mean  percentage | Maximum  percentage | Description |
| UV | % | 6 | 35.3 | 75 | Percentage understory vegetation* |
| MOSS | % | 0 | 33.5 | 75 | Percentage moss |
| BG | % | 0 | 36.4 | 563 | Percentage bare ground |
| TC | % | 0 | 0.57 | 1 | Percentage tree cover |
| UD | % | 6 | 19.8 | 61.7 | Percentage not accessible due to understory density of vegetation |

#### (*understory vegetation is defined as vegetation <100 cm that is either forbs, graminoids, and leaves on trees)
