# Supplementary material for: Large herbivores in novel ecosystems - Habitat selection by red deer (Cervus elaphus) in a former brown-coal mining area
Source: PLoS One. 2017 May 15;12(5):e0177431. doi: 10.1371/journal.pone.0177431 (PMC5432106; doi:10.1371/journal.pone.0177431)
Supplement: S1 Text — Granted copyright permission by COWI. (DOCX) [file pone.0177431.s005.docx]

Dear Anke,

COWI hereby grants the permission to use the 2 figures received by e-mail for the mentioned publication.

Med venlig hilsen/Best regards

Lars Flemming

Chief Market Manager DK

Head of Section

Mapping

COWI

COWI A/S (DK)

Parallelvej 2

2800 Kongens Lyngby

Denmark

Direct: +45 56 40 16 04

Phone: +45 56 40 00 00

Mobile: +45 51 56 10 48

Email: [lrfl@cowi.dk](mailto:lrfl@cowi.dk)

Sip: [lrfl@cowi.com](mailto:lrfl@cowi.com)

Website: [www.cowi.dk](http://www.cowi.dk) - [www.cowi.com](http://www.cowi.com)

LinkedIn   Facebook   Twitter

Print only if necessary

-----Original Message-----

From: Cowi

Sent: Wednesday, March 08, 2017 10:12 AM

To: Lars Flemming [<LRFL@cowi.com>](mailto:LRFL@cowi.com)

Subject: FW: copyright permission scientific publication

-----Original Message-----

From: Anke Müller [<mailto:anke.mueller@tum.de>]

Sent: Tuesday, March 07, 2017 6:12 PM

To: Cowi [<cowi@cowi.com>](mailto:cowi@cowi.com)

Subject: copyright permission scientific publication

Dear Sir or Madam,

when I was a Master student at Aarhus University (section for Ecoinformatics and Biodiversity) we conducted a scientific study we now want to publish in the peer-reviewed journal PLOS ONE. We would also like to include two figures for which we used orthophotos from Danmark (see attached) that were conducted by COWI. The journal now asks me and my co-authors (Prof. Jens-Christian Svenning is among them) to present a written permission from the copyright holder.

We therefore request permission for the open-access journal PLOS ONE to publish Figure 1 and Figure 2 (both attached to this mail) under the Creative Commons Attribution License (CCAL) CC BY 4.0 (<http://creativecommons.org/licenses/by/4.0/>). Please be aware that this license allows unrestricted use and distribution, even commercially, by third parties. Please reply and provide explicit written permission to publish Figure 1 and Figure 2 under a CC BY license.

We hope you will grant us the permission to use the orthophotos for our scientifc publication. We will have to hand in the written consent of your company and the following line will be added to each figure's

caption: “Reprinted from [ref] under a CC BY license, with permission from [name of publisher], original copyright [original copyright year].”

If you have any questions, please don't hesitate to contact me.

Yours sincerely,

Anke Müller (on behalf of all co-authors)
