# Supplementary material for: Large herbivores in novel ecosystems - Habitat selection by red deer (Cervus elaphus) in a former brown-coal mining area
Source: PLoS One. 2017 May 15;12(5):e0177431. doi: 10.1371/journal.pone.0177431 (PMC5432106; doi:10.1371/journal.pone.0177431)
Supplement: S2 Text — Granted copyright permission by ESRI. (DOCX) [file pone.0177431.s006.docx]

Hello Anke,

The TUM does own several licenses of our software. With it comes the general right to use our Basemaps either in one of our software products or as a screenshot in publications, presentations or marketing collaterals and so forth. For the latter there is only one thing you have to keep in mind: You have to cite Esri and its data providers either in the picture itself or near the picture. The following article in our online help describes in detail how this can be done easily: <http://doc.arcgis.com/en/arcgis-online/reference/static-maps.htm>

If you have any further questions please don't hesitate to ask us.

Best,

++

Kerstin van de Sand

Product Management Content

Esri Deutschland GmbH

Niederlassung Köln

Konrad-Adenauer-Ufer 41-45

50668 Köln

Telefon +49 89 207 005 1760

[k.vandesand@esri.de](mailto:k.vandesand@esri.de)

esri.de

Geschäftsführung: Jürgen Schomakers

Sitz: Kranzberg, HRB 186099, Amtsgericht München

Ust. IDNr. DE 271819187

++

-----Ursprüngliche Nachricht-----

Von: Anke Müller [<mailto:anke.mueller@tum.de>]

Gesendet: Dienstag, 7. März 2017 18:30

An: Reception Esri Kranzberg [<info@esri.de>](mailto:info@esri.de)

Betreff: copyright permission for scientific publication

Dear Sir or Madam,

when I was a Master student at Aarhus University (section for Ecoinformatics and Biodiversity) we conducted a scientific study we now want to publish in the peer-reviewed journal PLOS ONE. We would also like to include one figures for which we used ESRI ArcGIS Online Basemaps (see figure attached, small location maps on the right hand side). The journal now asks me and my co-authors (Prof. Jens-Christian Svenning is among them) to present a written permission from the copyright holder.

We therefore request permission for the open-access journal PLOS ONE to publish Figure 1 (attached to this mail) under the Creative Commons Attribution License (CCAL) CC BY 4.0 (<http://creativecommons.org/licenses/by/4.0/>). Please be aware that this license allows unrestricted use and distribution, even commercially, by third parties. Please reply and provide explicit written permission to publish Figure 1 under a CC BY license.

We hope you will grant us the permission to use the orthophotos for our scientifc publication. We will have to hand in the written consent of your company and the following line will be added to each figure's

caption: “Reprinted from [ref] under a CC BY license, with permission from [name of publisher], original copyright [original copyright year].”

If you have any questions, please don't hesitate to contact me.

Yours sincerely,

Anke Müller (on behalf of all co-authors)
